# Prognostic and clinicopathological significance of transcription factor c-Jun in hypopharyngeal squamous cell carcinoma: a 3-year follow-up retrospective study

**DOI:** 10.1186/s12885-022-10113-5

**Published:** 2022-09-26

**Authors:** Qiang Huang, Min Ye, Feiran Li, Lan Lin, Chunyan Hu

**Affiliations:** 1 Department of Otorhinolaryngology, Eye & ENT Hospital, Fudan University, Shanghai, 200031 China; 2 Department of Pathology, Eye & ENT Hospital Fudan University, Shanghai, 200031 China

**Keywords:** Prognosis, Clinicopathological significance, c-Jun, Head and neck cancer, Hypopharyngeal squamous cell carcinoma

## Abstract

**Purpose:**

To investigate the expression and prognostic value of c-Jun in hypopharyngeal squamous cell carcinoma (HPSCC).

**Methods:**

A retrospective study was performed on a cohort of 99 HPSCC patients. The expression of c-Jun and phosphorylated-c-Jun (p-c-Jun) was evaluated via immunohistochemistry (IHC) staining. Overall survival (OS) and progression-free survival (PFS) were assessed using Kaplan–Meier method and multivariate Cox regression analysis.

**Results:**

The high expression of c-Jun and p-c-Jun in HPSCC accounted for 60.61% and 16.16%, respectively. High expression of c-Jun was closely associated with cT stage (*p* = 0.0401), tumor size (*p* = 0.0276), number of lymph node metastases (*p* = 0.0205) and pathological differentiation (*p* = 0.0108). The expression of c-Jun^high^ (*p* = 0.0043), p-c-Jun^high^ (*p* = 0.0376) and c-Jun^high^/p-c-Jun^high^ were closely associated with poor OS. The Cox proportional multivariate hazard model revealed that lymphovascular invasion and c-Jun expression were independent influencing factors of OS in HPSCC patients.

**Conclusion:**

Our findings suggest that c-Jun is a reliable prognostic factors in HPSCC patients.

## Introduction

Hypopharyngeal squamous cell carcinoma (HPSCC) is one of the common squamous cell carcinomas of the head and neck, and 70 to 85% of cases are already in advanced stage when diagnosed due to the hidden location of the onset [1]. Cervical lymph node metastasis is an important factor affecting the prognosis of HPSCC [2]. Compared with patients without lymph node metastasis, the 5-year survival rate of patients with lymph node metastasis decreased by 50%, among which the 5-year survival rate of patients with single lymph node metastasis was 50%, and the 5-year survival rate of patients with bilateral metastasis was only 33% [3]. Therefore, it is crucial to identify effective and robust biomarkers to predict the prognosis of HPSCC patients.

The unbalanced expression of tumor suppressor genes and oncogenes is the basis for the occurrence and development of tumors [4]. c-Jun, a common transcription factor, is a major component of the dimeric transcription factor activator protein-1 (AP-1) which is a paradigm for transcriptional response to extracellular signaling [4, 5]. After stimulation, c-Jun can immediately express and generate transcription factors, regulate the transcription and expression of other genes, affect the growth, development and differentiation of normal cells, and lead to malignant transformation of cells [6, 7]. Evidence for the role of c-Jun in cancer has been established by quantifying the amount of c-Jun from various primary cancers tissue samples [8]. Xu et al. performed an integrative analysis of c-Jun prognostic value in oral squamous cell carcinoma (OSCC) through a multi-center cohort study, founding that high expressions of c-Jun was associated with poor prognosis and proved to be high risky predictors of death in OSCC [9]. Similar to their results, Eckhoff et al. showed that Jun proteins (pc-Jun and JunD) influence carcinogenesis and tumour progression, suggesting a significant role as prognostic predictors in human ovarian carcinoma [10]. Liu et al. found that c-Jun knockdown using siRNAs resulted in a significant declined induction chemotherapy IC50 in HPSCC cell line, identifying c-Jun as candidate genes that confer induction chemotherapy resistance, which would help in the discovery of potential therapeutic markers for HPSCC patients [11]. However, certain questions remain before its expression and independent prognostic significance of c-Jun in HPSCC can be stated.

Herein, we examined c-Jun, activated p-c-Jun, clinical information, and treatment outcomes in a range of HPSCC patients. To our knowledge, our study is the first to evaluate the expression and prognostic value of c-Jun in HPSCC patients.

## Methods and materials

### Patient tissue and ethics approval

A cohort of 99 formalin fixed paraffin-embedded (FFPE) HPSCC tissues were obtained from patients diagnosed with HPSCC pathologically after surgery from January 2015 to January 2018 from the Department of Otorhinolaryngology, Eye & ENT Hospital of Fudan University. All participants provided written informed consent forms. This study was approved by the Ethics Committee of Eye & ENT Hospital of Fudan University (No. 2018036).

### Surgical operation and postoperative adjuvant treatment

All patients received surgical therapies and were recommended for postoperative adjuvant treatment according to Guidelines of Chinese Society of Clinical Oncology (CSCO, version 2022) and National Comprehensive Cancer Network (NCCN) treatment guidelines (version 2.2016). Different surgical procedures were performed according to the location and invasion of the tumor, including partial hypopharyngectomy, partial laryngectomy + partial hypopharyngectomy, total laryngectomy + partial hypopharyngectomy, total laryngectomy + total hypopharyngectomy, and total laryngectomy + total hypopharyngectomy + partial or total esophagectomy. Several patients underwent surgical resection alone owing to their poor health status and personal unwillingness to undergo other treatment procedures. Postoperative adjuvant treatment included postoperative radiotherapy (pRT) and postoperative chemoradiotherapy (pCRT). pRT involved fractionation of 2 Gy/fraction once daily, five times a week. Meanwhile, pCRT was platinum-based concurrent CRT of cisplatin (45–50 mg/day) administered across three consecutive days [12].

### Immunohistochemical (IHC) staining and assessment

BenchMark Autostainer (Ventana Medical Systems, Tucson, USA) was used to perform IHC staining. Primary antibodies used in IHC staining are as followed: c-Jun (1:200, CST), Phospho-c-Jun (Ser73)(1:200, CST) and p53 (1:200, Gene Tech). Positive c-Jun and p-c-Jun were mainly cell nucleus staining. All sections were graded from level 0 to level 4 according to the following assessment: level 0, no positive cells; level 1, 1–24% positive cells; level 2, 25–49% positive cells; level 3, 50–74% positive cells; and level 4, ≥75% positive cells. Level 0 to level 2 was defined as low expression, and level 3 to level 4 was defined as high expression. The staining results were checked independently by two senior pathologists, and the discrepancies in immunostaining reviewing were solved by consensus.

### Detection of HPV genotype

Detection of HPV genotypes were analyzed by real-time polymerase chain reaction (PCR) using formalin fixed paraffin-embedded (FFPE) tumor samples. Briefly, after deparaffinization and rehydration, DNA was isolated from FFPE tissue using QIAamp DNA FFPE Tissue Kit (Qiagen, Valencia, CA, USA) in accordance with the manufacturer’s instructions. Real-time PCR amplifications were performed in a Thermal Cycler (ABI 7500 Real-Time PCR System, Life Technologies, Shanghai, China) using HPV Genotyping Real-time PCR Kit (Hybribio Limited, China) which is a real-time multiplex PCR test for the detection of 23 HPV genotypes (HPV16, 18, 31, 33, 35, 39, 45, 51, 52, 56, 58, 59, 66, 68, 6, 11, 42, 43, 44, 53, 81, 73 and 82), in accordance with the manufacturer’s instructions. Intracellular control DNA (β-globin DNA) was used to assess sample quality and PCR inhibitors. The HPV-positive tumor was defined as a tumor for which there was specific positive amplification of either HPV subtype.

### Statistical analysis

The endpoints of follow-up were overall survival (OS) and progression-free survival (PFS). OS was defined as the time from diagnosis to the last follow-up or death from any reason. PFS was calculated as time to recurrence or metastasis of HPSCC or the last follow up or death from any cause. Statistical analysis was performed using IBM SPSS Statistics (version 22.0; IBM, Armonk, NY, USA), and graphed using GraphPad Prism (version 8; GraphPad Software, La Jolla, CA). The Fisher’s exact test or chi-square test was performed for categorical variables. OS and PFS were evaluated using the Kaplan-Meier method and Gehan-Breslow-Wilcoxon test. The univariate and multivariate analyses were carried out with the Cox proportional hazards model. Differences were considered significant if the *P* value was < 0.05.

## Results

### Overview of main clinical features of the cohort

A cohort of consecutive 99 HPSCC patients was included in the current study from January 2015 to January 2018 in Eye & ENT Hospital, Fudan University. The median age at diagnosis was 59.36 ± 8.12 years, and 98.99% (1/99) of the patients were male. The tumors mainly originated from the pyriform sinus (*n* = 86, 86.87%), followed by the postcricoid region (*n* = 7, 7.07%) and the posterior pharyngeal region (*n* = 6, 6.06%). According to the 8th AJCC staging system, 5.05% (5/99) patients were in early stage of HPSCC (T1–2N0) and 94.95% (94/99) patients were in locoregionally advanced stages (T3–4 and T1–4N+). Meanwhile, no cervical lymph node metastasis (N0) was found in 15.15% of the patients (15/99). Cervical lymph node metastases (N1-N3) were reported in 84.85% of patients (84/99).

According to the location and invasion of the tumor, different surgical procedures were performed. Three patients (3.03%) had partial hypopharyngectomy, 44 patients (44.44%) had partial laryngectomy + partial hypopharyngectomy, 37 patients (37.38%) had total laryngectomy + partial hypopharyngectomy, six patients (6.06%) had total laryngectomy + total hypopharyngectomy, and nine patients (9.09%) had total laryngectomy + total hypopharyngectomy + partial or total esophagectomy. Patients were recommended for postoperative adjuvant treatment according to guidelines of CSCO and NCCN treatment guidelines. A total of 69 patients (72.63%) received pCRT, while 26 patients (27.37%) received pRT alone. Four patients (4.04%) received surgery treatment alone without postoperative adjuvant therapy for their own reasons. There were 5 patients (5.05%) with tumor cell infiltration in the surgical margin, and the R0 resection rate was 94.95%.

The tumors were well to well-moderately differentiated in 9.09% patients (9/99), and were moderately to moderately-poorly differentiated in 90.91% patients (90/99). There are 15.15% (15/99) patients positive in HPV infection status. During the follow-up period, 16.16% (16/99) patients developed regional tumor recurrence and 18.18% (18/99) patients developed distant metastases. The overall 3-year survival rate of the cohort was 76.77%. The mean overall survive time and progression-free survive time was 23.89 ± 8.85 and 21.86 ± 10.12 months, respectively.

### Correlation between c-Jun expression and clinicopathological variables

**Fig. 1:**
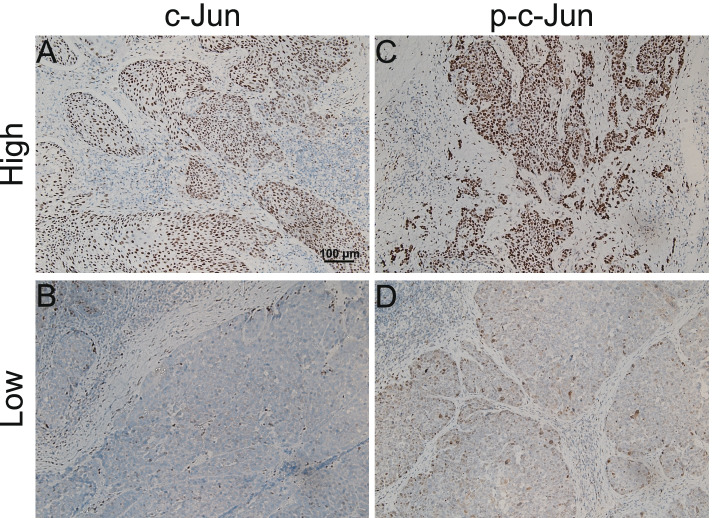
Representative immunohistochemical staining images of c-Jun and p-c-Jun in HPSCC patients **A** high expression level of c-Jun; **B** low expression level of c-Jun; **C** high expression level of p-c-Jun; **D** low expression level of p-c-Jun. Scale bar:100 μm

The expression of c-Jun and p-c-Jun was mainly located in the tumor nucleus in HPSCC tissue via immunohistochemical analysis. In our study, the high expression of c-Jun in HPSCC accounted for 60.61% patients. Meanwhile, 16.16% patients had high expression of p-c-Jun (Fig. 1). As shown in Table 1, the expression level of c-Jun was significantly correlated with cT stage (*p* = 0.0401), tumor differentiation (*p* = 0.0108), tumor size (*p* = 0.0276) and number of lymph node metastases (*p* = 0.0205). Furthermore, we found that the expression level of p-c-Jun was significantly correlated with number of lymph node metastases (*p* < 0.0001) and thyroid gland invasion (*p* = 0.0344).

**Table 1:** Association between clinicopathological characteristics with the expression of c-Jun and p-c-Jun in 99 patients with hypopharyngeal squamous cell carcinoma

Variable	c-Jun			p-c-Jun		
	Low	High	*p* value	Low	High	*p *value
**Age (years)**			0.7826			0.1051
≤ 60	21	34		43	12	
> 60	18	26		40	4	
**Smoking**			0.3084			0.3451
No	12	13		23	2	
Yes	27	47		60	14	
**Drinking**			0.0754			0.6287
No	14	18		26	6	
Yes	42	25		57	10	
**Tumor site**			0.2371			0.6869
Pyriform sinus	36	50		71	15	
Not pyriform sinus	3	10		12	1	
**cT stage**			0.0401*			0.0627
T1–2	23	24		36	11	
T3–4	15	37		47	5	
**cN stage**			0.3917			0.4534
N0	4	11		14	1	
N1–3	35	49		69	15	
**Clinical stage**			> 0.9999			0.5889
I + II	2	3		5	0	
III + IV	37	57		78	16	
**pT stage**			0.2343			0.0615
T1–2	19	22		31	10	
T3–4	20	38		52	6	
**pN stage**			> 0.9999			0.2031
N0	4	7		11	0	
N1–3	35	53		72	16	
**Pathological stage**			0.7935			0.7545
I + II + III	10	14		21	3	
IV	29	46		62	13	
**Tumor differentiation**			**0.0108†**			> 0.9999
Well+ well-moderately	0	9		8	1	
Moderately+ moderately-poorly	39	51		75	15	
**Surgical margin status**			0.1742			0.2558
≥ 0.5 cm	16	33		39	10	
< 0.5 cm	23	27		44	6	
**Tumor size (cm)**			0.0276*			0.7960
≤ 3.5	26	33		49	10	
> 3.5	9	31		34	6	
**Lymph nodal fusion**			0.1974			0.2646
No	26	47		63	10	
Yes	13	13		20	6	
**Number of lymph node metastases**			**0.0205†**			**< 0.0001†**
≤ 1	36	44		68	12	
> 1	3	16		4	15	
**Metastatic lymph node size (cm)**			0.1183			0.3868
≤ 3	22	43		56	9	
> 3	17	17		27	7	
**Cervical nodal necrosis**			0.6438			0.0509
No	22	31		48	5	
Yes	17	29		35	11	
**Lymphovascular invasion**			> 0.9999			0.1577
No	36	54		77	13	
Yes	3	6		6	3	
**Extracapsular spread**			0.1522			0.1400
No	27	49		66	10	
Yes	12	11		17	6	
**Fixation of hemilarynx**			0.1764			0.0798
No	21	24		34	11	
Yes	18	36		48	6	
**Thyroid gland invasion**			0.6455			**0.0344**†
No	38	56		80	14	
Yes	1	4		2	3	
**Laryngeal invasion**			0.6306			0.7285
No	15	26		35	6	
Yes	24	34		48	10	
**Ki-67 expression**			0.0534			0.9513
≤ 30%	17	38		46	9	
> 30%	22	22		37	7	
**HPV infection status**			0.2303			0.6611
No	31	53		71	13	
Yes	8	7		12	3	

* *p* value was tested from Chi-square test. † *p* value was tested from Fisher’s exact test

**Fig. 2:**
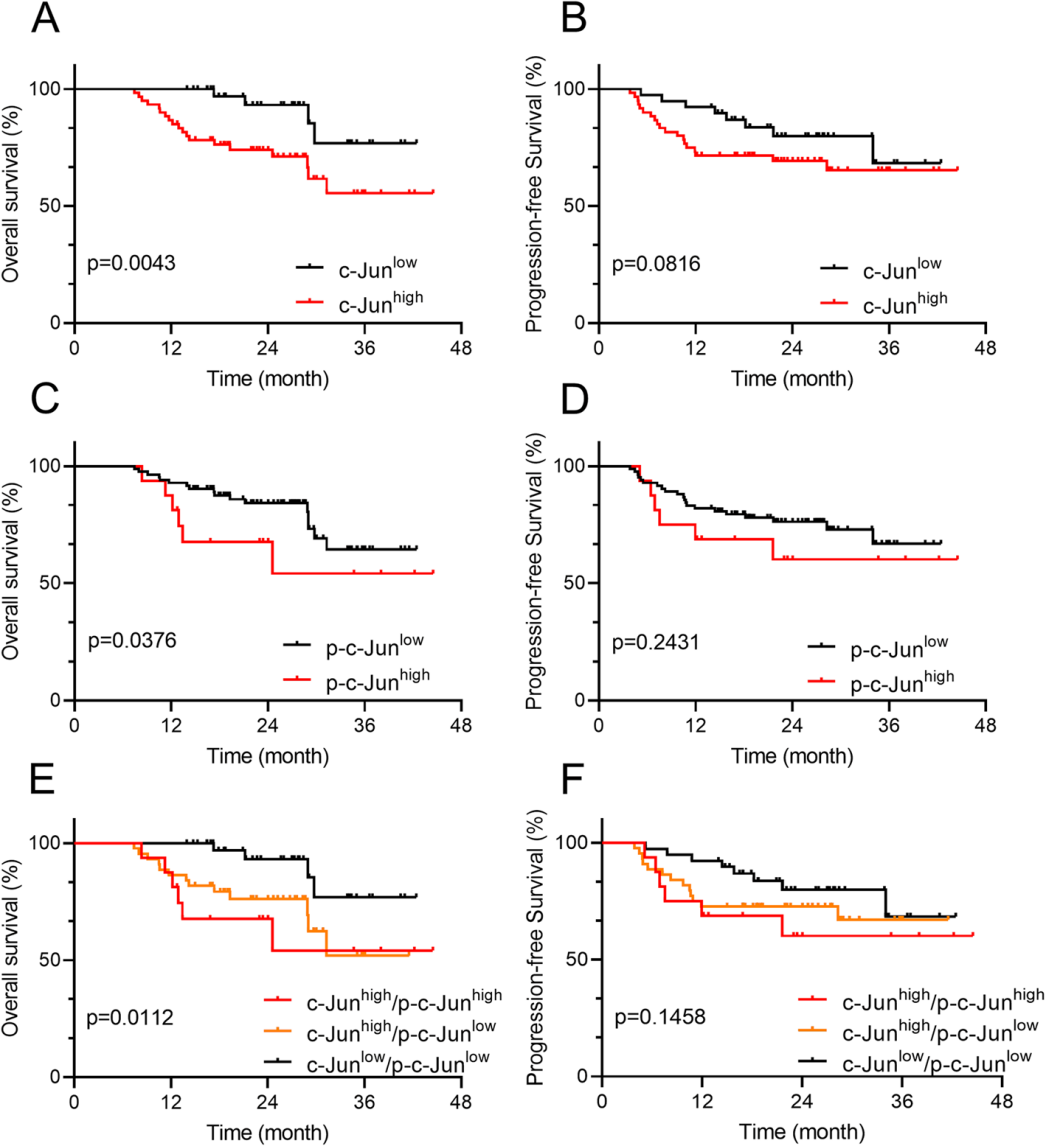
Survival curves of 99 HPSCC patients with different expression of c-Jun and p-c-Jun OS (**A**,** C**) and PFS (**B**, **D**) according to the expression level of c-Jun and p-c-Jun, suggesting that OS in HPSCC patients with high c-Jun (*n* = 60 cases) or p-c-Jun expression (*n* = 16 cases) is significantly shorter than those with low c-Jun (*n* = 39 cases) or p-c-Jun (*n* = 83 cases) expression, but there was no significant difference in PFS. **E**, **F** OS and PFS according to the grouped expression level of c-Jun/p-c-Jun (red line, *n* = 16 cases; orange line, *n* = 44 cases; black line, *n* = 39 cases). The survival curves were defined by the Kaplan–Meier method, the tests of survival rates were performed by Gehan-Breslow-Wilcoxon test

Then, we analyzed the prognostic significance of c-Jun and p-c-Jun in HPSCC patients by Kaplan-Meier survival analysis. It’s turned out that the mean OS and PFS time were 25.56 and 23.49 months in the c-Jun low expression group; and were 22.80 and 20.80 months in the c-Jun high expression group. And the mean OS and PFS time were 24.17 and 22.09 months in the p-c-Jun low expression group; and were 22.44 and 20.70 months in the p-c-Jun high expression group. As shown in Fig. 2A, B, the 3-year OS rate in the c-Jun high expression group was significantly lower than that in the c-Jun low expression group (68.33% vs 89.74%, *p* = 0.0043). Similarly, the 3-year OS rate in the p-c-Jun high expression group was significantly lower than that in the p-c-Jun low expression group (62.50% vs 79.52%, *p* = 0.0376). However, neither c-Jun nor p-c-Jun had significant prognostic significance for PFS in HPSCC patients (Fig. 2C, D). In order to reduce the confounding factors of a single indicator, c-Jun and p-C-Jun were subgrouped to study their prognostic significance. We found that the OS of the c-jun^high^/p-c-jun^high^ group was the worst, while the OS of the c-jun^low^/p-c-jun^low^ group was the best, and the OS of the c-jun^high^/p-c-jun^low^ group was in the middle (*p* = 0.0112, Fig. 2E). Nevertheless, the subgroup had significant no prognostic significance for PFS (*p* = 0.1458, Fig. 2F).

**Fig. 3:**
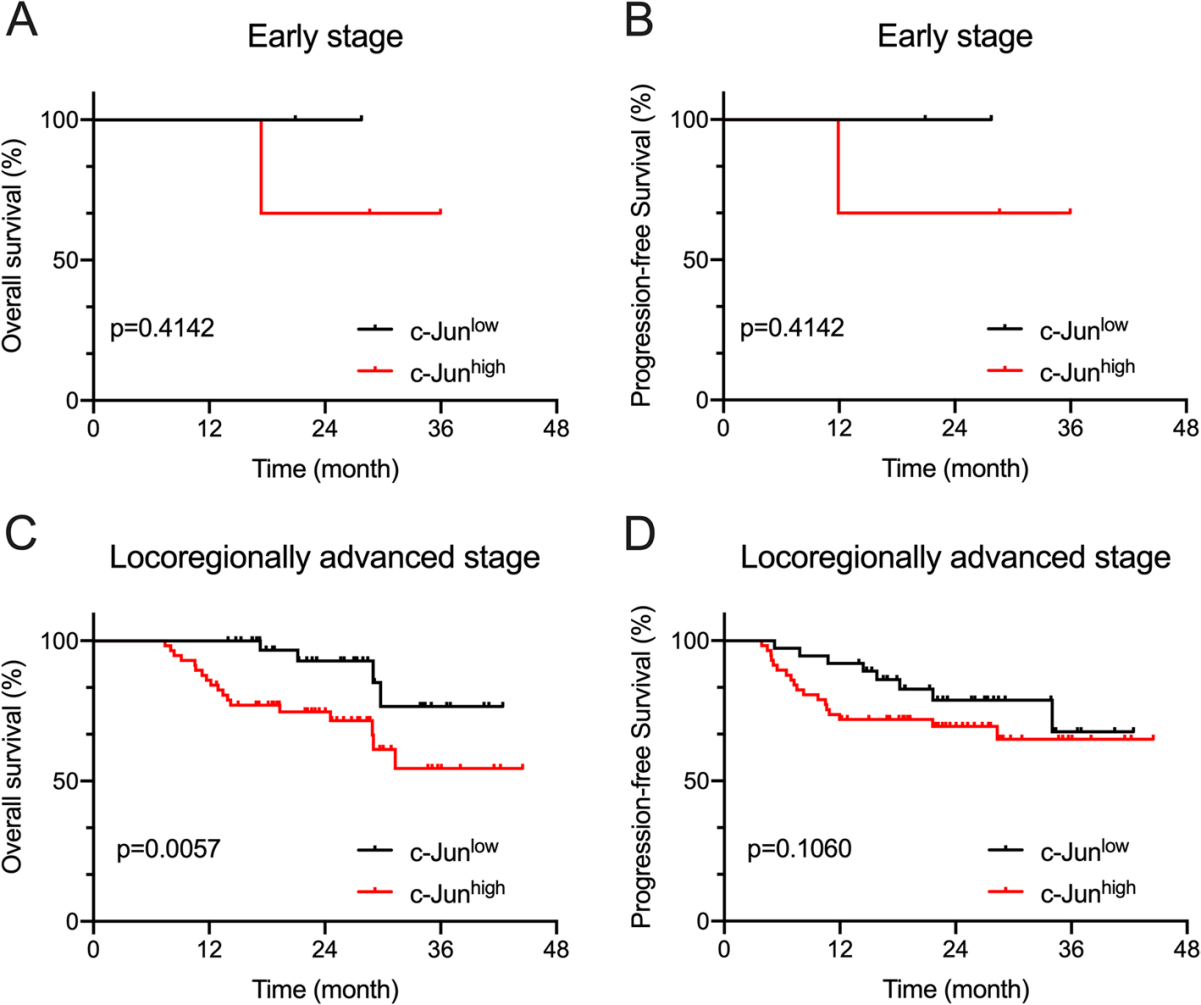
Survival curves of early and locoregionally advanced stage HPSCC patients with different expression of c-Jun OS (**A, C**) and PFS (**B, D**) according to the expression level of c-Jun, suggesting that OS in locoregionally advanced stage HPSCC patients with high c-Jun (*n* = 57 cases) is significantly shorter than those with low c-Jun (*n* = 37 cases) expression, but there was no significant difference in PFS and early stage patients. The survival curves were defined by the Kaplan–Meier method, the tests of survival rates were performed by Gehan-Breslow-Wilcoxon test

Then, the c-Jun expression of early (T1–2N0) and locoregionally advanced stage (T3–4 and T1–4 N+) patients was grouped. As a result, the number of patients included in the early patients group was insufficient to effectively analyze the prognostic role of c-Jun in early patients group (*p* = 0.4142 for OS and PFS, Fig. 3A, B). However, in the locoregionally advanced stage group, c-Jun expression was proved to be a potent indicator of OS (*p* = 0.0057, Fig. 3C) but not PFS (*p* = 0.1060, Fig. 3D).

### High c-Jun expression were independent influencing factors of OS

To identify predictive factors of OS and PFS in HPSCC patients, univariate analysis of various prognostic factors was performed. We found that tumor site (*p* = 0.043, HR: 0.382, 95% CI: 0.150–0.972) and c-Jun expression (*p* = 0.019, HR: 3.626, 95% CI: 1.233–10.665) were influencing factors of OS in HPSCC patients. Furthermore, tumor site (*p* = 0.018, HR: 0.353, 95% CI: 0.149–0.837), cT stage (*p* = 0.041, HR: 2.367, 95% CI: 1.036–5.410), pathological stage (*p* = 0.021, HR: 10.477, 95% CI: 1.420–77.295) and lymphovascular invasion (*p* = 0.029, HR: 2.971, 95%CI: 1.120–7.879) were influencing factors of PFS in HPSCC patients (Table 2). Moreover, multivariate cox regression analysis was conducted for all variables significantly correlated with OS and PFS in univariate analysis (Table 3), among which lymphovascular invasion (*p* = 0.036, HR: 3.464, 95% CI: 1.084–11.075) and c-Jun expression (*p* = 0.035, HR: 3.313, 95% CI: 1.089–10.081) were independent influencing factors of OS. Pathological stage (*p* = 0.038, HR: 8.339, 95% CI: 1.119–62.132) and lymphatic vascular invasion (*p* = 0.020, HR: 3.367, 95% CI: 1.213–9.345) were independent influencing factors of PFS.

**Table 2:** Univariate analysis of various prognostic factors in 99 patients with hypopharyngeal squamous cell carcinoma

Variable	Overall Survival		Progression-free Survival	
	HR (95% CI)	p	HR (95% CI)	p
**Age (years)**				
≤ 60	Reference	0.082	Reference	0.057
> 60	0.454 (0.186–1.107)		0.448 (0.196–1.024)	
**Smoking**				
No	Reference	0.418	Reference	0.576
Yes	0.701 (0.296–1.658)		0.789 (0.345–1.806)	
**Drinking**				
No	Reference	0.993	Reference	0.907
Yes	0.996 (0.421–2.355)		0.953 (0.428–2.123)	
**Tumor site**				
Pyriform sinus	Reference	**0.043**	Reference	**0.018**
Not pyriform sinus	0.382 (0.150–0.972)		0.353 (0.149–0.837)	
**cT stage**				
T1–2	Reference	0.218	Reference	**0.041**
T3–4	1.717 (0.727–4.054)		2.367 (1.036–5.410)	
**cN stage**				
N0	Reference	0.724	Reference	0.541
N1–3	0.823 (0.279–2.427)		1.454 (0.437–4.837)	
**Clinical stage**				
I + II	Reference	0.826	Reference	0.660
III + IV	1.252 (0.169–9.302)		1.565 (0.212–11.540)	
**pT stage**				
T1–2	Reference	0.248	Reference	0.073
T3–4	1.688 (0.694–4.105)		2.197 (0.928–5.202)	
**pN stage**				
N0	Reference	0.298	Reference	0.215
N1–3	2.901 (0.390–21.576)		3.535 (0.479–26.075)	
**Pathological stage**				
I + II + III	Reference	0.107	Reference	**0.021**
IV	2.722 (0.807–9.184)		10.477 (1.420–77.295)	
**Tumor differentiation**				
Well+ well-moderately	Reference	0.784	Reference	0.440
Moderately+moderately-poorly	0.815 (0.190–3.505)		0.622 (0.186–2.078)	
**Surgical margin status**				
≥ 0.5 cm	Reference	0.145	Reference	0.311
< 0.5 cm	0.537 (0.233–1.240)		0.674 (0.315–1.445)	
**Tumor size (cm)**				
≤ 3.5	Reference	0.840	Reference	0.462
> 3.5	1.089 (0.476–2.490)		1.328 (0.624–2.830)	
**Lymph nodal fusion**				
No	Reference	0.696	Reference	0.965
Yes	0.820 (0.303–2.216)		0.981 (0.414–2.323)	
**Number of lymph node metastases**				
≤ 1	Reference	0.550	Reference	0.531
> 1	1.356 (0.500–3.678)		1.338 (0.539)	
**Metastatic lymph node size (cm)**				
≤ 3	Reference	0.141	Reference	0.147
> 3	1.870 (0.813–4.301)		1.758 (0.821–3.764)	
**Cervical nodal necrosis**				
No	Reference	0.561	Reference	0.604
Yes	1.275 (0.563–2.890)		1.221 (0.573–2.600)	
**Lymphovascular invasion**				
No	Reference	0.064	Reference	**0.029**
Yes	2.793 (0.944–8.268)		2.971 (1.120–7.879)	
**Extracapsular spread**				
No	Reference	0.943	Reference	0.704
Yes	0.964 (0.358–2.601)		1.182 (0.499–2.799)	
**Fixation of hemilarynx**				
No	Reference	0.617	Reference	0.330
Yes	1.239 (0.535–2.873)		1.474 (0.675–3.220)	
**Thyroid gland invasion**				
No	Reference	0.841	Reference	0.603
Yes	0.814 (0.109–6.076)		1.467 (0.346–6.223)	
**Laryngeal invasion**				
No	Reference	0.866	Reference	0.817
Yes	0.929 (0.395–2.185)		1.098 (0.499–2.416)	
**c-Jun expression**				
Low	Reference	**0.019**	Reference	0.185
High	3.626 (1.233–10.665)		1.748 (0.765–3.996)	
**p-c-Jun expression**				
Low	Reference	0.129	Reference	0.311
High	2.060 (0.809–5.242)		1.601 (0.645–3.976)	
**Ki-67 expression**				
≤ 30%	Reference	0.454	Reference	0.858
> 30%	0.721 (0.305–1.701)		1.072 (0.501–2.291)	
**HPV infection status**				
No	Reference	0.927	Reference	0.995
Yes	0.945 (0.279–3.204)		1.003 (0.345–2.915)	

*HR* hazard ratio, *CI* confidence interval at the 95% level

**Table 3:** Multivariate cox regression analyses of prognostic factors associated with overall survival and progression-free survival in 99 patients with hypopharyngeal squamous cell carcinoma

Variable	Overall Survival		Progression-free Survival	
	HR (95% CI)	p	HR (95% CI)	p
**Tumor site**				
Pyriform sinus	Reference	0.160	Reference	0.051
Not pyriform sinus	0.481 (0.173–1.336)		0.394 (0.154–1.004)	
**cT stage**				
T1–2	Reference	0.772	Reference	0.239
T3–4	1.149 (0.450–2.934)		1.686 (0.707–4.019)	
**Pathological stage**				
I + II + III	Reference	0.227	Reference	**0.038**
IV	2.174 (0.617–7.662)		8.339 (1.119–62.132)	
**Lymphovascular invasion**				
No	Reference	**0.036**	Reference	**0.020**
Yes	3.464 (1.084–11.075)		3.367 (1.213–9.345)	
**c-Jun expression**				
Low	Reference	**0.035**	Reference	0.476
High	3.313 (1.089–10.081)		1.374 (0.573–3.295)	

*HR* hazard ratio, *CI* confidence interval at the 95% level

## Discussion

In this current study, we evaluated the expression of c-Jun and phosphorylated-c-Jun (p-c-Jun) via IHC staining, founding that high expression of c-Jun was closely associated with the clinicopathological variables in HPSCC patients. More importantly, c-Jun expression was independent influencing factor of OS in HPSCC patients.

Transmission of the extracellular signals through the cytoplasm is mediated by cascades of protein kinases, ultimately leading to phosphorylation of transcription factors and activation of downstream genes [5]. Phospho-c-Jun are relatively unstable and exist only transiently in the cells [5], which may partially explain the expression of p-c-Jun in HPSCC tissue was lower than the expression of c-Jun. In our results, we found that the OS of the c-jun^high^/p-c-jun^high^ group was the worst, while the OS of the c-jun^low^/p-c-jun^low^ group was the best, and the OS of the c-jun^high^/p-c-jun^low^ group was in the middle when c-Jun and activated p-c-Jun were subgrouped to study their prognostic significance. Wang et al. reported that c-jun, c-fos and p53 alone were not associated with the OS in OSCC patients. However, the co-expression of c-jun/c-fos/p53 was identified as independent prognostic factors for overall survival. Simultaneous co-expression of these markers in OSCC patients might prove to be a useful risk indicator [13]. Furthermore, high expressions of c-Jun or Fra-1 were associated with poor prognosis in OSCC patients, meanwhile the high expression of Fra-1 meant worse prognosis of patients than the high expression of c-Jun. Besides, the interaction effect of c-Jun and Fra-1 was antagonism, when the expression of c-Jun and Fra-1 was both high, the HR was lower than the hazard ratio when only the Fra-1 was at high expression [9]. It is possible that they all compete to bind to the AP-1 site or form “inactive” heterodimers at high expression [14]. Therefore, the combination analysis of c-Jun with genes associated with c-Jun promoting or antagonistic pathways may explain the prognostic significance of patients more than c-Jun or p-c-Jun alone.

There is a compelling evidence of the human papilloma virus including HPV16 E6 oncogene drives cell transformation and oncogenic processes of head and neck cancer [15, 16]. It’s reported that interaction between matrix hyaluronan (HA) and CD44 (an HA receptor) promotes c-Jun phosphorylation followed by phospho-c-Jun nuclear translocation and co-localization with HPV16 E6 in the nucleus of HPV+ head and neck cancer cells [17]. These results suggested that c-Jun may interact with HPV to promote tumor progression. In this current study, the overall prevalence of HPV infection was 15.15% and the high-risk HPV types were the most frequently identified, which does not allow excluding HPV as a risk factor in HPSCC patients. However, when relating c-Jun and p-c-Jun expression and HPV infection, no statistically significant relationship is observed which was consistent with the results reported by Acay et al. in OSCC [18]. Furthermore, Gupta et al. reported that c-Jun participated only in HPV negative and poorly differentiated tongue cancer [19], which contradicts our findings that c-Jun was distributed in all types of HPV infection status and pathological differentiation. We hypothesized that the possible reasons for the discrepancy included the sample size and the anatomic site in head and neck cancer patients.

To further validated the results displayed by current study, studies with larger sample size of HPSCC patients are required. To fully understand the specific mechanism by which c-Jun promotes HPSCC, more in vivo and in vitro experimental studies are required. And it will be valuable to perform the longitudinal study to explore whether the expression of c-Jun would be changed during the tumor progression.

Collectively, c-Jun could be valuable prognostic biomarkers in HPSCC, and may help to provide a new sight for the studies of tumor prognosis and tumor treatment in HPSCC.

## Data Availability

The datasets generated and/or analyzed during the current study are not publicly available due to privacy and ethical issues, but are available from the corresponding author on reasonable request.
